# The Management of Classical Hodgkin's Lymphoma: Past, Present, and Future

**DOI:** 10.1155/2011/865870

**Published:** 2011-04-06

**Authors:** S. E. Richardson, C. McNamara

**Affiliations:** Department of Haematology, Royal Free Hospital, Pond Street, London NW3 2TB, UK

## Abstract

The management of classical Hodgkin's lymphoma (CHL) is a success story of modern multi-agent haemato-oncology. Prior to the middle of the twentieth century CHL was fatal in the majority of cases. Introduction of single agent radiotherapy (RT) demonstrated for the first time that these patients could be cured. Developments in chemotherapy including the mechlorethamine, vincristine, procarbazine and prednisolone (MOPP) and Adriamycin, bleomycin, vinblastine and dacarbazine (ABVD) regimens have resulted in cure rates of over 80%. Even in relapse, CHL patients can be salvaged with high dose chemotherapy and autologous haematopoietic stem cell transplantation (ASCT). Challenges remain, however, in finding new strategies to manage the small number of patients who continue to relapse or progress. In addition, the young age of many Hodgkin's patients forces difficult decisions in balancing the benefit of early disease control against the survival disadvantage of late toxicity. In this article we aim to summarise past trials, define the current standard of care and appraise future developments in the management of CHL.

## 1. Introduction

The management of classical Hodgkin's lymphoma (CHL) is a success story of modern multiagent haemato-oncology. Prior to the middle of the twentieth century CHL was fatal in the majority of cases. Introduction of single-agent radiotherapy (RT) demonstrated for the first time that these patients could be cured. Developments in chemotherapy including the mechlorethamine, vincristine, procarbazine, and prednisolone (MOPP) and adriamycin, bleomycin, vinblastine, and dacarbazine (ABVD) regimens have resulted in cure rates of over 80%. Even in relapse, CHL patients can be salvaged with high-dose chemotherapy and autologous haematopoietic stem cell transplantation (ASCT).

Challenges remain, however, in finding new strategies to manage the small number of patients who continue to relapse or progress. In addition, the young age of many Hodgkin's patients forces difficult decisions in balancing the benefit of early disease control against the survival disadvantage of late toxicity. In this paper we aim to summarise past trials, define the current standard of care, and appraise future developments in the management of CHL.

## 2. Clinical Risk Stratification

Accurate assessment of prognosis is essential to direct appropriate therapy at the earliest opportunity. Current practice is to define risk groups on adverse presenting clinical risk factors such as disease stage, presence of B symptoms, bulky disease, and patient age. Using these parameters, research groups have defined three treatment groups requiring different treatment intensities (Table 1). In addition, clinical markers can be used to create an International Prognostic Score (IPS) for advanced stage disease (Table 2) [1].

## 3. Management of Early Stage Favourable CHL

Limited stage CHL is a highly curable disease. In 1989 an international workshop and symposium met in Paris to review the outcomes of CHL cases treated between the 1960s and 1987. More than 9000 early stage patients were reviewed from a total of 14702 cases. Long-term mortality was 22%; in the first decade this was mainly due to relapse, but after 13 years of followup deaths from second malignancies and cardiovascular disease were relatively high. The most important risk factors for late mortality were older age and exposure to wide-field radiotherapy or MOPP-like chemotherapy [2]. This was the first acknowledgment that late effects of treatment could affect long-term survival; the relatively young age of CHL patients results in late effects having a disproportionate impact. Trials in early stage CHL have therefore tried to progressively reduce exposure to toxic agents.

Monotherapy with radiotherapy (RT) is an effective treatment in early stage CHL, but wide radiation fields and high doses are associated with considerable late toxicity, and it is no longer standard practice. Major trials in early stage favourable CHL (Table 3) have investigated reducing RT field size and dose and incorporating abbreviated chemotherapy regimens into combined modality treatment (CMT).

### 3.1. Combined Modality Therapy

An early study by the Southwest Oncology Group compared subtotal lymphoid irradiation (STLI) with CMT (STLI plus adriamycin/vinblastine; the least toxic agents of the ABVD regimen used in advanced stage CHL) [3]. The trial was closed early after a significant benefit in failure-free survival (FFS) emerged in the CMT arm. Overall survival (OS) was not different at 3 years. Haematological toxicity was significantly higher in the CMT group. The German Hodgkin Lymphoma Study Group (GHSG) compared extended-field (EF) RT with CMT (2ABVD and EF RT) in the HD7 trial [4]. Seven-year followup showed improved freedom from treatment failure (FFTF) in the CMT arm at the expense of more haematological toxicity, but no difference in OS or second malignancy. The EORTC H7F trial compared STLI to CMT consisting of 6 EBVP (epirubicin, bleomycin, vinblastine, and prednisolone) and involved-field (IF) RT [5]. EBVP was thought to confer less cardiac toxicity and nausea compared to ABVD. Ten-year event-free survival (EFS) was better in the CMT arm with a very low rate of secondary malignancies, but OS was again not different. Soon after, the EORTC/GELA H8F trial demonstrated that both improved 10-year EFS and OS when comparing CMT (3MOPP/ABV and IF RT) to STLI monotherapy [6]. The Milan group directly compared radiation fields within ABVD-based CMT in a single centre study of STLI versus IF RT. With 12-year followup there was no difference in disease progression or survival, but the study was not powered to detect noninferiority of IF RT to STLI [7].

### 3.2. Chemotherapy as Monotherapy

The consensus opinion from these trials was that CMT provides better disease control than radiotherapy alone and that reduction in radiotherapy to IF RT was feasible. Two to four cycles of ABVD were established as the chemotherapy regimen of choice. This led other groups to question the benefit of RT and study chemotherapy monotherapy.

The NCI-C/ECOG H6 study combined favourable and unfavourable (age >40, ESR >50 mm/h, >3 sites of disease, mixed cellularity/lymphocyte-deplete histology) early stage CHL into groups receiving some radiotherapy or chemotherapy alone [8]. One hundred and twenty three favourable risk patients were randomised to STLI or 4–6 cycles of ABVD. No differences in 5-year freedom from progression (FFP) or OS were seen. A single-centre study by Straus et al. randomised 152 patients to CMT (6ABVD + RT) or 6 cycles of ABVD alone [9]. Five-year PFS and OS were not different, but the study was powered to detect an expected benefit of over 20% for CMT rather than equivalence between the two therapies. A systematic review by Herbst et al. showed benefit in tumour control and OS from CMT [10].

### 3.3. Current Standard of Care and Future Directions

The GHSG HD10 trial has defined the current standard of care for patients with early stage, favourable HL [11]. Using a 2 × 2 factorial design the group randomised 1370 patients to either 2 or 4 cycles of ABVD followed by either 20 or 30 Gray (Gy) IF RT. Five-year results show no difference in disease control between the two groups. There was less acute toxicity and fewer acute toxic deaths with 2 cycles of chemotherapy compared with 4 and less toxicity (e.g., mucositis, dysphagia) with 20 Gy compared with 30 Gy. The EORTC/GELA/IIL intergroup have an ongoing trial of 3 cycles of ABVD with IF RT. We believe, however, the current standard of care for early stage favourable CHL to be 2 cycles of ABVD followed by 20 Gy IF RT. 

Ongoing trials in early favourable CHL are looking at further reducing toxicity. The GHSG HD13 trial is investigating whether individual chemotherapeutic agents can be omitted from the ABVD regimen by comparing 2 cycles of ABVD, AV, ABV, or AVD followed by 30 Gy IF RT. Preliminary reports suggest a poorer outcome in patients not receiving dacarbazine. The EORTC H9F trial investigates the optimal dose of IF RT by randomising patients to 6 cycles of EBVP followed by 0, 20, or 36 Gy of IF RT. Until the results of H9F study are available a reasonable approach is 20 Gy of radiotherapy, in light of the GHSG HD10 results mentioned above. Future studies will define whether functional imaging can eliminate the need for radiotherapy altogether in early stage disease. The UK RAPID trial is a phase III multicentre randomised study assessing the role of FDG-PET imaging in patients with clinical stage IA/IIA Hodgkin's lymphoma. Patients who are PET negative after 3 cycles of ABVD are randomised to receive either involved-field RT (Arm A) or no further treatment (Arm B). Individuals who are PET positive will receive a further (4th) cycle of ABVD followed by involved-field radiotherapy. The GHSG HD16 trial randomises patients to either 2 cycles of ABVD plus 20 Gy IF RT and end-of-treatment PET or 2ABVD followed by RT in PET+ and observation in PET−.

## 4. Management of Early Stage Unfavourable CHL

It is clear that certain risk factors (Table 1) confer additional risk to early stage CHL that requires more intensive CMT treatment. Trials have sought to find the combination of chemotherapy and radiation exposure that provide optimal disease control whilst limiting toxicity (Table 4).

CMT is the established treatment for early stage unfavourable CHL. The outcome of the Canadian HD6 trial (above) was dominated by the unfavourable group benefiting from CMT (2ABVD plus STLI) compared to 4–6 cycles of ABVD alone (5-year FFP 95% versus 88%, *P* = .004) [8]. ABVD-based CMT has been shown to be superior to MOPP or EBVP in two well-conducted randomised controlled trials in this setting [5, 12]. The EORTC H8U trial tested 4 versus 6 cycles of MOPP/ABV and 36 Gy IF RT versus STLI demonstrating no difference [6]. The GHSG HD8 trial also looked at reducing radiation field from extended-field (EF) to IF RT following 4 cycles of ABVD-based chemotherapy [13]. In 1064 patients 5-year freedom from treatment failure (FFTF) and OS were equivalent, with less grade III-IV haematological toxicity in the IF RT arm.

### 4.1. Current Standard of Care and Future Directions

The results of the EORTC H8U and GHSG HD8 studies establish 4 cycles of ABVD-based chemotherapy followed by 36–40 Gy IF RT as the current standard of care in early stage unfavourable CHL.

Ongoing trials are looking at reducing exposure to radiation and testing more intensive chemotherapy regimens. The EORTC H10 trial is comparing CMT to chemotherapy alone. The GHSG HD 11 trial has a 2 × 2 design comparing 4 cycles of ABVD to BEACOPP (bleomycin, etoposide, adriamycin, vincristine, procarbazine, and prednisolone) followed by IF RT at either 20 or 30 Gy. Interim results suggest inferiority of 20 Gy compared to 30 Gy. The EORTC H9U trial also looks at increasing chemotherapy comparing 4 and 6 cycles of ABVD and 4 cycles of BEACOPP all followed by 30 Gy IF RT. The GHSG HD 14 trial compares 4ABVD or 2BEACOPP+2ABVD followed by 30 Gy IF RT. Interim results show a 6% superior PFS in the BEACOPP arm. With increasing interest in the prognostic value of interim positron emission tomography (PET) scanning (see later), the H10 EORTC/GELA/IIL intergroup study is assessing if a negative PET scan after two cycles of chemotherapy can be used to de-escalate from 6 to 4 cycles of ABVD. The GHSG HD17 trial treats patients with 2 cycles of escalated BEACOPP and 2ABVD followed by a randomisation based on PET if PET+ patients are randomised to 30 Gy RT by either IF or involved node and if PET− patients are randomised to observation or 30 Gy IF RT. 

## 5. Management of Advanced Stage HL

In the 1970s MOPP chemotherapy became the standard of care in advanced CHL with 20-year OS of 48%. Toxicity was high, however, with 100% infertility in men and a 2% rate of secondary leukaemia [14]. Through the 1980s and 1990s ABVD-based regimens were compared to MOPP, although the two regimens were never tested head-to-head in a randomised controlled trial (Table 5). In summary, alternating MOPP/ABVD and ABVD was equivalent and both were superior to MOPP alone [15, 16]. Alternating MOPP/ABVD was equivalent to MOPP/ABVD [17] or MOPP/ABV hybrid [18], but there was more toxicity in the MOPP/ABV hybrid arm. Duggan found that ABVD alone was equivalent to MOPP/ABV hybrid, but with reduced acute haematological toxicity and secondary AML/MDS. ABVD is therefore considered equivalent or possibly more efficacious than MOPP, but significantly less toxic [19].

### 5.1. Increasing Efficacy with Higher Intensity Regimens

Several groups have tried to develop more intensive chemotherapy regimes, by either escalating doses and number of drugs or reducing the dosing interval.

#### 5.1.1. BEACOPP-Based Regimens (Bleomycin, Etoposide, Doxorubicin (Adriamycin), Vincristine (Oncovin), Procarbazine, and Prednisolone)

The GHSG developed the BEACOPP regimen based on mathematical modelling to increase dose density and intensity [20]. Three variants have been trialled: standard BEACOPP (SB), escalated BEACOPP (EB), and BEACOPP-14, which is accelerated over a 14-day cycle. In the EB regimen doses of cyclophosphamide, etoposide, and doxorubicin are increased by 192%, 200%, and 140%, respectively. The seminal trial of SB and EB was the GHSG HD9 study [21]. 1196 patients were randomised between a baseline arm of 8 cycles of alternating COPP (cyclophosphamide replacing mechlorethamine in the MOPP regimen)/ABVD and SB or EB. 10-year FFTF and OS showed an advantage for treatment with EB [22]. Acute toxicity was higher with significantly more haematological and infectious toxicity, but no difference in nonrelapse mortality (NRM) of 7%. Secondary leukaemias/MDS were increased in both BEACOPP regimens, and infertility was universal.

This important trial provided for the first time a more effective, but toxic, regimen that could be used to improve the outcomes for those at highest risk of relapse. Interestingly EB benefited all IPS groups implying that this would not be a good method of identifying those most likely to benefit. The use of EB has not become widespread, however, as a number of criticisms can be made of the study. The control arm would not be considered to be the standard of care in most centres and the outcomes for ABVD alone have improved compared to historical comparisons. In addition, COPP/ABVD was given over a median of 46.3 days compared to a planned period of 30 days. AVBD, in contrast, is routinely delivered on time. In the HD9 study the more intensive SB and EB regimens were given, on time, over a median of 24.3 and 24.7 days, respectively. A large confirmatory multicentre RCT comparing SB/EB and modern, on-time ABVD alone is eagerly awaited by many who are concerned that the acute toxicity, infertility, and late malignancy associated with BEACOPP outweigh the benefits of early disease control.

Despite promising results, some patients continue to relapse. Patients with the highest IPS scores (4–7) treated with EB fare better than with COPP/ABVD, but still have a 30% 10-year mortality. Another group with a poor prognosis that does not respond to dose escalation is the elderly. The GHSG HD9_elderly_ study randomised 26 patients to alternating COPP/ABVD and 42 patients to SB [23]. EB was deemed too toxic to study, and the COPP/ABVD arm was shut early due to the results of HD9. Despite the COPP/ABVD arm having more unfavourable patients, no benefit to 5-year FFS (46%) or OS (50%) was demonstrated. Toxicity in the SB arm was high; only 38% of patients received the intended dose, TRM was 21% compared to 8% in the COPP/ABVD arm, and the incidence of any grade III-IV toxicity was 87% compared to 44% in COPP/ABVD.

The only other randomised comparison of a BEACOPP regimen comes from the Italian GISL HD 2000 trial [24]. In this study 4 cycles of BEACOPP (2EB, 2SB) were compared to another intensive regimen CEC (cyclophosphamide, lomustine, vindesine, melphalan, prednisolone, epidoxorubicin, vincristine, procarbazine, vinblastine, and bleomycin COPPEBVCAD) and a control group treated with ABVD. Only 81 patients were randomised to BEACOPP, but it did show a significantly better 5-year FFS. There was no difference in CR or OS, partly due to salvage treatments. BEACOPP had more grade III-IV haematological and infectious toxicity, but it was less toxic than reported in the GHSG HD9 study.

#### 5.1.2. Strategies to Reduce BEACOPP Toxicity

A number of groups are investigating strategies to capitalise on the effectiveness of EB, whilst minimising toxicity. Most strategies at present are attempting to minimise the number of EB cycles, either by protocol or interim PET assessment of response.

The GHSG HD12 trial employs a 2-by-2 design testing 8 × EB or 4 × EB/4 × SB followed by consolidation RT or observation [25]. Interim results of 1571 patients showed no difference in 5-year PFS or OS. Other GHSG studies investigating BEACOPP include the HD15 (8 × EB versus 8 × SB versus 6 × SB with a completion PET to decide on consolidation RT) and HD18 (2 × EB then escalate or de-escalate based on a PET scan, see later). The concept of mixing EB and SB is supported by an interim report of 321 patients treated by the Italian Intergroup showing better 3-year FFP in 4 × EB-4SB compared to 6–8 cycles of ABVD (87% versus 71%, *P* = .01) [26].

A second strategy under evaluation by the GHSG is to time-intensify BEACOPP into 14 days reducing the cumulative doses of agents that cause acute toxicity and secondary malignancy. Phase II data on 94 patients treated with 8 cycles of BEACOPP-14 by the GHSG showed 3-year rates of CR, FFTF, and OS of 94%, 97%, and 90%, respectively. 75% of cases experienced grade III-IV leukopenia resulting in a 12% rate of serious infection [27].

#### 5.1.3. Other Intensive Regimens

The Stanford group developed a 12-week regimen to time intensify certain agents and de-escalate/omit more toxic agents (Stanford V: doxorubicin, vinblastine, mechlorethamine, vincristine, bleomycin, etoposide, and prednisolone). Integral to this regimen was radiotherapy to any bulky disease (>5 cm), macroscopic splenic disease, or persistent disease by CT criteria after treatment. Pilot studies showed encouraging results with 5-year FFP and OS of 85–89% and 96%, respectively [28, 29]. An Italian Intergroup trial of 355 patients randomised to Modified Stanford V, MOPPEBVCAD, or ABVD showed disappointing results for modified Stanford V, but the aim of the study was to minimise radiotherapy exposure which undermined the strategy of the Stanford protocol [30]. A UK NCRI study of 520 patients randomised to ABVD or Stanford V with as protocol RT showed no difference in FFS/OS [31]. 

Another UK study, the LY09 trial, also failed to show any benefit of a multidrug regimen (ChlVPP/PABIOE versus ChlVPP/EVA ± RTX, see Table 6 for details) over ABVD [32].

### 5.2. Consolidation Treatment

#### 5.2.1. Consolidation Radiotherapy

Patients with poor risk features such as bulky disease or residual lesions by CT or PET criteria might be potential candidates for consolidation radiotherapy. This approach has the advantage of being less toxic than ASCT. The EORTC investigated 739 patients treated with MOPP/ABV chemotherapy [33]. If patients achieved CR (*n* = 512), they were randomised to IF RT or observation. Those achieving PR (*n* = 227) received RT. 8-year EFS and OS were the similar in each group suggesting a benefit for IF RT in the PR group. No difference in the rate of second malignancies was noted. The GELA H89 trial randomised patients to chemotherapy with either 6 × MOPP/ABV or ABVPP [34]. Those achieving CR or PR received consolidation with 2 further cycles of chemotherapy (*n* = 208) or STLI (*n* = 210). RT was not superior to chemotherapy consolidation. The GHSG HD15-PET study investigated patients treated with BEACOPP with residual disease >2.5 cm [35]. These cases proceeded to have a PET and were given RT on panel decision. PET-positive cases had a 1-year PFS of 96% compared to 86% if PET negative (*P* = .011). End of treatment PET positivity predicted for relapse within one year, despite additional radiotherapy. The above trials and a systematic review by Loeffler have contributed to abandon consolidation RT for patients in CR after chemotherapy [36]. 

A systematic review of the sensitivity and specificity of interim PET in advanced lymphoma identified 360 cases of advanced CHL in 13 studies [37]. Interim PET showed a sensitivity of 0.81 (95% CI 0.72–0.89) and specificity of 0.97 (95% CI 0.94–0.99) indicating that an approach of targeted IF RT based on PET is a reasonable consolidation strategy in selected patients failing to achieve CR.

#### 5.2.2. Autologous Stem Cell Transplant

Consolidation high-dose therapy and ASCT of patients at very high risk of relapse has been investigated by some groups in small studies. The Scotland and Newcastle Lymphoma Group HD3 trial identified 65 “highest-risk” patients based on age, stage, lymphocyte count, baseline haemoglobin, and presence of bulky disease [38]. Patients were given 3 cycles of PVACE-BOP chemotherapy and then randomised to either ASCT or 2 further cycles of chemotherapy. No differences in 6-year time-to-treatment failure (TTF) (79% versus 85%) or OS were found. The European Intergroup identified “unfavourable” cases based on 2 of high LDH, large mediastinal mass, >1 extranodal site, low haematocrit, or inguinal involvement [39]. Those achieving PR/CR after 4 cycles of ABVD were randomized to 4 more cycles of ABVD (*n* = 80) or ASCT (*n* = 83). 5-year FFS and OS were not significantly different. Consensus opinion is therefore that ASCT in first CR is not indicated.

## 6. Late Effects

With the advent of chemotherapy regimens that can increase disease control at the expense of late effects, risk stratification is rapidly being incorporated into standard practice. When considering the risks of late effects it is important to distinguish between relative (RR) and absolute (AR) risk and in particular how this affects long-term survival.

### 6.1. Second Malignancy

Solid malignancies account for the majority** (**59–80%) of second malignancies. Younger patients have a higher RR due to their low baseline risk, whereas older patients have a higher AR particularly for carcinoma of the lung. Risk is related to exposure to both chemotherapy and RT, but the risk from chemotherapy seems to plateau.

The RR of haematological malignancies, in particular acute myeloid leukaemia (AML) and myelodysplastic syndrome (MDS), is significantly raised during the first decade of followup. ABVD appears to confer a relatively small risk of AML/MDS compared to MOPP- or BEACOPP-based regimens [19, 22, 30].

### 6.2. Fertility

ABVD chemotherapy appears to have relatively mild effects on fertility. A small study of 36 women treated with 4–6 cycles of ABVD showed that 70% could conceive within 1 year compared to 75% of controls [40]. Data from the EORTC H6-9 study of 355 males with early stage HD looked at elevation of follicle stimulating hormone (FSH) as a marker of gonadal damage [41]. FSH was elevated in 3% after RT, 8% of men treated with chemotherapy containing no alkylating agents and 60% of men treated with regimens such as MOPP or BEACOPP that contain alkylators. At 19 months FSH returned to baseline in 82% of those not exposed to alkylating agents, compared with only 30% treated with alkylating agents. The GHSG has also demonstrated 100% azoospermia or dyspermia in 38 males treated with 8 cycles of SB or EB, although interestingly only 23% were normozoospermic prior to starting chemotherapy. Median time to recovery was 3.6 years [42]. A small GHSG study of ovarian protection in 23 women treated with EB using either the oral contraceptive or a GnRH analogue showed no benefit of either intervention [43]. 12-month levels of antimullerian hormone (AMH) were reduced in all with a 0% ovarian follicle preservation rate (95% CI 0–12%).

### 6.3. Cardiovascular

Data on late effects on the cardiovascular system are less complete, but a range of adverse outcomes have been reported including coronary artery disease (CAD), left ventricular dysfunction (LVD), valvular stenoses, pericardial disorders, and other vascular diseases such as carotid or subclavian artery stenoses. Relative risks of dying from CAD are increased by 5 with an absolute risk of 10–12% at 15–25 years. This risk is disproportionately increased by the presence of conventional cardiac risk factors, which should be optimised. RR of symptomatic valvular disease requiring valve replacement is 8-9 times higher with an absolute risk of 6% at 20 years.

### 6.4. Pulmonary

Rates of bleomycin lung toxicity have been reported to be as high as 30% in the US intergroup study and were related to age and prior RT [18]. Rates of fatalities are approximately 1.5–2% [18, 31]. Some are questioning the value of bleomycin in the ABVD regimen, and this was supported by a retrospective review showing reasonable outcomes in 40 patients discontinuing bleomycin due to dyspnoea [44].

## 7. Risk Adapted Treatment

Various strategies have been employed by cooperative groups to stratify patients into risk groups at presentation so as to target initial treatment intensity. Clinical scores such as the IPS are in widespread use, but interestingly the benefit of EB was spread across all IPS risk groups. A number of biomarkers have been evaluated including morphology, immunophenotype (e.g., CD15, CD20), apoptotic proteins (e.g., Bcl-2), T-cell composition, measuring tumour burden (e.g., B2MG, soluble CD30), or in situ EBV. None of these is currently in widespread clinical use.

### 7.1. Assessing Chemosensitivity by Interim PET

Initial response to chemotherapy has been investigated as a prognostic marker. A large Italian/Danish observational study of 260 patients studied the prognostic value of a PET after 2 cycles (PET2) of chemotherapy [45]. Cases that were PET positive after 2 cycles (19%) had a 2-year FFS of 12.8% compared to 95% in the PET negative group. PET2 was predictive of outcome across IPS scores, and, in a multivariate analysis PET2, stage IV and age over 45 were the most significant prognostic markers. Concerns have been raised, however, about the uniformity of PET reporting.

Dann et al. [46] published a small, nonrandomised study of risk-adapted therapy based on PET2 results. 108 patients were given 2 cycles of SB (IPS 1-2) or EB (IPS 3–7) and then underwent PET assessment. PET-positive patients received 4 cycles of EB and PET negative 4 cycles of SB. 69 cases started with SB of which 14%  (*n* = 10) were escalated to EB after PET2. 39 cases started with EB, and 79%  (*n* = 31) were de-escalated to SB. Only 7 cases received 6 cycles of EB. There were no differences in 5-year PFS (85%) or OS (90%) between the groups. Interestingly, PET2 had a high negative predictive value (98%) for progression, but a surprisingly low positive predictive value (27%), suggesting a beneficial effect of intensive treatment in the higher-risk patients. Another observation in this study was that 9 of 10 cases that were escalated had residual PET positivity at the end of treatment (including radiotherapy consolidation). In 7 of 9 cases this regressed over 14 months, and these patients remain in CR. 

Ongoing phase III studies of interim PET scanning include the UK RATHL and GHSG HD18 studies. RATHL treats patients with 2 cycles of ABVD and then has a double randomisation: if PET2-positive patients are escalated to either BECOPP-14 or EB, if PET2-negative patients receive either ABVD or AVD. HD18 starts patients with 2EBs and then randomises PET2+ patients to 6EB with or without rituximab and PET2-negative patients to 2 or 6 cycles of EB.

#### 7.1.1. Current Standard of Care and Future Directions

The current standard of care in advanced Hodgkin's lymphoma is contentious. Most clinicians continue to support 6–8 cycles of ABVD as standard. EB is an option in patients at higher risk, but many await a confirmatory phase III trial. PET positivity after 2 cycles of chemotherapy is emerging as a powerful predictive tool, and studies are investigating if escalation or de-escalation of therapy after PET2 benefits high- or low-risk cases, respectively. Using end-of-treatment PET scanning to direct consolidation RT may be beneficial to those not achieving CR.

Even in advanced stage disease, however, the majority of patients will achieve long-term disease control. Efforts to improve OS by limiting toxicity include limiting number of cycles of chemotherapy, removing more toxic drugs such as bleomycin or doxorubicin, and incorporating new, less toxic agents into current regimens. Trials in this area will have to report progressively longer followups if true reductions in late toxicity are to be proven.

## 8. Relapsed/Refractory

Approximately 5–10% of cases will suffer primary refractory disease defined as no response or progression within 90 days of treatment, and a further 10–30% will relapse [47]. These patients have a poor prognosis if treated with conventional chemotherapy alone. In a single-centre historical review of 107 patients Longo et al. [48] demonstrated a CR rate of 49%, but this was durable in only 5–10%, and median survival was 16 months. Those relapsing after one year (late relapses) had a significantly improved CR and OS rate (80% and 25%) compared to those with early relapses (50% and 10%) despite high rates of secondary AML in the survivors. Another review of 115 relapsed or refractory patients treated with MOPP/ABVD + RT showed 8-year rates of FFSP and OS to be 22% and 28% in early relapses and 44% and 54%, late relapse [49]. Those with primary progressive disease had the worst prognosis (FFSP 0% and OS 8%).

## 9. High-Dose Chemotherapy and ASCT

2 randomised controlled trials have established the benefit of high-dose chemotherapy and ASCT in relapsed and refractory CHL. A UK BNLI study randomising 40 patients to mini-BEAM or BEAM ASCT showed 3-year EFS of 10% versus 53% in the ASCT arm (*P* = .025) [50]. No difference in OS was found at the early followup. A larger EMBT/GHSG randomised 161 chemosensitive patients having received 2 cycles of salvage dexa-BEAM to either 2 further cycles or BEAM ASCT [51]. 3-year FFTF was better in the ASCT arm (55% versus 34%, *P* = .019) with no difference in OS, although a large number of deaths were noted during all cycles of dexa-BEAM.

Whether ASCT benefits chemorefractory cases remains unclear. Registry data such as the Seattle series of 64 patients have shown encouraging results (5-year PFS 17% and OS 31%), but current practice is to maximise overall response during salvage chemotherapy [52].

### 9.1. Tandem ASCT

Some groups have advocated the use of tandem ASCT in very high-risk cases. In 43 patients with induction failure or very-unfavourable relapse 2-year OS was 65% (0% if no ASCT, 40% if 1 ASCT, and 74% if tandem ASCT) [53]. The nonrandomised GELA H96 trial treated 150 high-risk patients (primary refractory or 2 of relapse within 1 year, relapse in radiation field, and stage III/IV at relapse) with tandem ASCT compared with 95 cases with intermediate risk relapse (one of the above risk factors) who underwent single ASCT [54]. Fewer than 70% of high-risk patients tolerated both transplants, but outcomes were promising with 5-year freedom from second failure (FF2F) of 73% and 46% and OS of 85% and 57% in intermediate- and high-risk arms, respectively. 

A high-dose sequential approach (2 cycles of DHAP, cyclophosphamide + GCSF and stem cell collection followed by methotrexate and vincristine, etoposide, and BEAM conditioned ASCT versus conventional ASCT is under investigation by the GHSG [55].

## 10. Salvage Chemotherapy Regimens

With the evidence indicating improved outcomes in chemosensitive patients undergoing ASCT, the overall response rate (ORR) of salvage chemotherapy and ability to subsequently harvest stem cells are critical. No randomised trials have compared salvage regimens; results of phase II studies are compared in Table 7, and overlapping confidence intervals suggest no major differences in ORR [56–66]. Pending more information on efficacy, the choice of salvage chemotherapy is therefore based on expected tolerability and the treating centre's experience.

## 11. Salvage Radiotherapy

Salvage treatment with radiotherapy may be appropriate in selected cases. The GHSG retrospective analysed 100 patients treated with RT alone at disease progression. 5-year FFTF was 29% and OS 51% with an ORR of 81%. This was worse in advanced stage, early relapse, or poor performance score patients [67].

## 12. Allogeneic Stem Cell Transplantation

### 12.1. Myeloablative Allografting

Treatment-related mortality (TRM) of multiple relapsed CHL patients treated with myeloablative allogeneic stem cell transplant (AlloSCT) is prohibitive at 48%–61% (Table 8) [68–72]. Even in patients treated earlier in their disease at the Johns Hopkins Oncology Center, total TRM was 42% with a 10-year PFS of only 26% [71]. An EBMT study matched 45 cases of AlloSCT to ASCT demonstrating a TRM of 48% versus 27%  (*P* = .0411) that rose to 65 versus 12%  (*P* = .0054) in chemosensitive patients. Myeloablative AlloSCT is therefore not recommended in relapsed CHL [69].

### 12.2. Reduced Intensity Conditioned (RIC) Allografting

RIC AlloSCTs offer a strategy to reduce TRM but require graft versus lymphoma (GvL) activity for disease control. Evidence for GvL activity can come from increased relapse rates after T-cell depletion, the association of graft-versus-host disease (GvHD) with reduced relapse rates, and most convincingly responses to donor leukocyte infusions [73].

Published outcomes of RIC strategies in CHL are summarised in Table 9 [74–78]. An EBMT study comparing 89 RIC to 79 myeloablative AlloSCTs showed an improved 3-year TRM of 24% versus 48%  (*P* = .003) and significantly better 5-year OS [78]. An association of chronic GvHD to reduced risk of relapse was noted, but conditioning intensity was also important with total body irradiation being associated with adverse outcome. It is important that steroid treatment of GvHD does not confound the reporting of disease responses to DLI. Studies by Alvarez et al. [76], Peggs et al. [75], and Anderlini et al. [79] have shown an association between DLI and the development of GvHD with durable disease responses in approximately 50% of cases. Interestingly T-cell depletion with alemtuzumab has been shown to reduce chronic GVHD, without increasing relapse rate leading some to suggest that this may be due to a reduction in T-regulator cell inhibition of antitumour cytotoxic T cells [80].

A UK study compared 38 RIC AlloSCTs with 34 matched historical controls that had responded to salvage treatment and survived for one year and hence were deemed fit enough to undergo RIC [81]. 5-year current PFS and 10-year OS from diagnosis were 42% and 48% in the RIC arm compared to 18%  (*P* = .075) and 15%  (*P* < .0001) in the control group. 5-year NRM was a more favourable 19%, and DLI responses were seen in 8 of 15 patients.

### 12.3. Current Standard of Care and Future Directions

High-dose therapy and autologous stem cell rescue is established as the treatment of choice in relapsed and refractory CHL. Outcomes are significantly better if patients have chemosensitive disease prior to transplant [82]. A number of salvage regimens exist, but they have not been compared in randomised controlled trials; overlapping confidence intervals suggest no major differences in effectiveness.

Allogeneic transplantation as a therapeutic option for patients with second relapse or refractory disease who respond to chemotherapy is an exciting therapeutic manoeuvre that continues to be studied. Although myeloablative protocols are associated with prohibitive toxicity, RIC transplantation offers lower TRM and the possibility of prolonged DFS in multiple relapsed patients, particularly using DLI. Randomised trials are needed to define which patients would benefit most, to identify optimal conditioning, and to evaluate the importance of response to salvage chemotherapy on outcome.

## 13. Experimental Therapies

Due to the success of conventional treatments in managing HL, the number of patients available to trial novel agents is limited. Clinical circumstances that particularly merit their investigation include multiple relapse/refractory patients, those PET+ after salvage therapy and the elderly.

### 13.1. Monoclonal Antibodies

Following the success of rituximab in CD20 positive non-Hodgkin lymphoma, a number of differentially expressed CHL cell surface markers have been evaluated as targets for monoclonal antibody (mAb) therapy (Table 10).

#### 13.1.1. CD30

CD30 is densely expressed and highly restricted on CHL cells, existing as a membrane-bound and a soluble form. Early trials of anti-CD30 mAbs were disappointing due to poor binding, poor activation of immune response, and quenching by soluble CD30 [83, 84]. Strategies to improve antibody performance have included designing mAbs to be selective for the membrane form and humanising the antibody. One promising strategy is by-passing antibody-mediated cellular cytotoxicity to conjugate the mAb to an antimicrotubule agent (SGN-35). Early trials of SGN-35 have shown promising ORRs of between 39 and 47% with updated results expected imminently [85, 86]. The AETHERA trial is investigating SGN-35 maintenance for patients at high risk (chemoresistant, early relapse, and extranodal disease) after autologous SCT.

#### 13.1.2. CD25

CD25 is the interleukin-2 receptor that is overexpressed on CHL cells. An ^131^I radio-immunoconjugate (CHT-25) showed promising results in a phase I study on 12 CHL patients with single photon emission CT showing tumour-specific uptake [87]. Of 9 patients treated with >1200 MBq/m^2^ 3 achieved CR and 3 PR. Delayed myelotoxicity was the most common adverse event with a platelet and neutrophil nadir at 38 and 53 days, respectively.

Other surface markers being investigated as CHL selective targets include CD40, TRAIL, IL13 signalling, and CD 80.

### 13.2. Intracellular Signalling Pathways

#### 13.2.1. Histone Deacetylase (HDAC) Inhibitors

Epigenetic modification of gene expression is deranged in a wide variety of malignancies, and histone deacetylase enzymes (HDACs) are crucial mediators of this process. Small molecule class I and pan HDAC inhibitors have been developed, and phase I trials in relapsed and refractory CHL have shown ORR of between 4% and 54% with mild toxicity [88–91].

#### 13.2.2. PI3K/Akt/mTOR

The PI3K/Akt/mTOR signalling pathway is one of the most frequently deranged in all malignancies. Phase II data on 19 CHL patients treated with the mTOR inhibitor everolimus showed an ORR of 47% with mild haematological toxicity [92]. The redundancy of many intracellular signalling pathways suggests that combinations of small molecule signalling modulators will be required to improve efficacy.

#### 13.2.3. Anti-NFkB and Bortezomib

Another key signalling molecule of CHL is the transcription factor NFkB, which is constitutively activated in many lymphomas. Cytoplasmic IkB usually inhibits NFkB from translocating to the nucleus and upregulating multiple pro-proliferative genes. In CHL various aberrant signalling pathways converge to cause IkB to be degraded in the proteasome.

The feasibility of proteasome inhibitors to restore IkB inhibition of NFkB has been demonstrated in preclinical studies by Zheng et al. [93]. Proteasome inhibition in four CHL cell lines demonstrated antiproliferative activity even in the presence of mutated IkB or CD30, CD40 and RANK receptor activation. Cytotoxic activity of gemcitabine was also increased and bortezomib synergised with anti-CD30 antibody 5F11 in preclinical studies [94], however, no significant single-agent activity has been seen in early clinical trails in relapsed HL [95, 96]. It may be that bortezomib finds a role either as a means of sensitising CHL cells to cytotoxic agents or by synergising with other small molecule inhibitors of intracellular signalling.

#### 13.2.4. HSP90

Other intracellular targets under investigation include the heat shock proteins such as HSP90.

### 13.3. Microenvironment/Immunotherapy

It has long been noted that the majority of cells in CHL lymph nodes are reactive. Hodgkin Reed-Sternberg (HRS) cells are difficult to grow in culture, and it has been postulated that they are dependent on a supportive microenvironment to proliferate. Therapeutic interventions to modify this interaction include strategies to deplete supporting cells or modulate intercellular interactions.

#### 13.3.1. Anti-CD20 (Rituximab)

Promising early results have been demonstrated with the anti-CD20 mAb rituximab, despite CD20 being poorly expressed by HRS cells. Possible mechanisms include altering the nodal microenvironment or direct targeting of a putative Hodgkin stem cell. 22 patients (6 with CD20+ CHL) treated with rituximab at the MD Anderson Center had an ORR of 22%, regardless of CD20 expression [97].

In a phase II study by the GHSG in CHL with more than 30% malignant cells expressing CD20 the ORR was as high as 86% with 75% of responses durable at 1 year [98].

Adding rituximab to chemotherapy has been tested in small studies. In combination with ABVD as first-line treatment 52 patients had an encouraging 3-year EFS and OS of 82% and 100% [99]. In the relapsed and refractory setting adding rituximab to gemcitabine in 33 patients produced a 48% ORR [100]. The GHSG HD18 trial is randomising PET2-positive patients to either EB or R-EB, and we await these results with interest.

#### 13.3.2. Lenalidomide

Lenalidomide is a thalidomide analogue with a number of immunomodulatory and antiangiogenic properties. It has been postulated to interfere with the cancer-microenvironment interaction, and two studies in relapsed CHL have shown ORRs of 50–53% despite some significant dose reductions [101, 102].

## 14. Conclusion

The current standard of care of CHL depends on disease stage and risk. We believe that early favourable CHL should be treated with 2 cycles of ABVD chemotherapy and 20 Gy IF RT according to the GHSG HD10 study. The results of the EORTC H8U and GHSG HD8 studies establish 4 cycles of ABVD-based chemotherapy followed by 36–40 Gy IF RT as the current standard of care in early stage unfavourable CHL. 6–8 cycles of ABVD are the current standard in advanced stage CHL, but we expect a rapidly increasing role for BEACOPP-based regimens in high-risk cases guided by interim PET responses. Conversely we believe the use of toxic agents such as bleomycin or radiotherapy will become more restricted in lower-risk patients.

High-dose therapy with ASCT is the standard of care for relapsed and refractory CHL. Every effort should be made to achieve chemosensitivity prior to transplant. With the demonstration of a GvL effect, RIC-alloSCT is a promising area of research that may benefit carefully selected patients at risk of recurrent relapse.

Hodgkin's lymphoma research has resulted in major advances in outcomes for patients by the targeted use of effective, standardised therapies employed according to reliable prognostic factors. Although CHL can be cured in the majority of cases with conventional chemotherapy, further improvements in efficacy and reduction in toxicity will rely on the development of the above and other promising novel agents and their incorporation into individualised combinations.

## Figures and Tables

**Table 1:** Risk groups in CHL: clinical criteria used by European Organisation for Research and Treatment of Cancer (EORTC) and German Hodgkin Lymphoma Study Group (GHSG) to define treatment groups. Abbreviations: ESR, erythrocyte sedimentation rate (mm/h); CS, clinical stage; MTR, mediastinum-to-thorax ratio.

	EORTC	GHSG
Risk factor (RF)	(1) Age ≥ 50 y	(1) Large mediastinal mass
	(2) B symptoms + ESR:	(2) Extranodal disease/massive spleen
	(a) No symptoms + ESR ≥ 50	(3) ESR
	(b) Symptoms + ESR ≥ 30	(a) >50 without B symptoms
	(3) >3 nodal areas	(b) >30 with B symptoms
	(4) MTR ≥ 0.35	(4) >3 involved nodal regions
Early stage without RFs	CS I-II	CS I-II
Early stage unfavourable/ intermediate stage	CS I-II with one RF	CS I or IIA with RF
		CS IIB with RF 3 or 4
Advanced stage	CS III-IV	CS IIB with RF 1 or 2
		CS III or IV

**Table 2:** Outcome for advanced Hodgkin's lymphoma based on International Prognostic Score. Risk factors: age >45 y, stage IV, male sex, white cell count >15 × 10^9^/L, lymphocyte count <0.6 × 10^9^/L/8%, albumin <40 g/L, and haemoglobin <10.5 g/dL.

	5 year	
Number of factors	Freedom from progression (%)	Overall survival (%)
0	84	89
1	77	90
2	67	81
3	60	78
4	51	61
5 or more	42	56

**Table 3:** Trials of treatments for early stage favourable Hodgkin's lymphoma. Numbers in bold are statistically significant with *P* values where significant. Abbreviations: OS: overall survival; EF RT: extended-field radiotherapy; IF-RT: involved-field radiotherapy; STLI: subtotal lymphoid irradiation; Gy: gray; ABVD: doxorubicin, bleomycin, vinblastine, and dacarbazine; AV: doxorubicin, vinblastine; EBVP: epirubicin, bleomycin, vinblastine, and prednisolone; MOPP/ABV: mechlorethamine, vincristine, procarbazine, prednisolone, doxorubicin, bleomycin, and vinblastine; SWOG: Southwest Oncology Group; GELA: Group d'Etudes des Lymphomes le l'Adulte; NCIC: National Cancer Institute of Canada; ECOG: Eastern Co-operative Oncology Group; MSKCC: Memorial Sloan Kettering Cancer Center.

Treatment regimen (author/trial)	No. of Pts	Outcome (%, measure, time)	OS (%)	Haem toxicity (% Gr III-IV)	All acute toxicity (% grade III-IV)	Second malignancy (haem/solid, %)
STLI (36–40 Gy)	161	81 (FFS 3 y)	>95%	42	—	—
3AV + STLI (36–40 Gy)	165	**94**	>95%	**57**	—	—
(Press SWOG 2001)		*P* < .001		*P* = .004		

EF RT 30 Gy (+10 Gy IF RT)	311	67 (FFTF 7 y)	92	0.8	—	3.2
2ABVD + EF RT 30 Gy (+10 Gy IF RT)	316	**88**	94	14.8	—	2.2
(Engert GHSG-HD7 2007)		*P* < .001				

STLI	165	78 (EFS 10 y)	92	—	—	1/2
6EBVP + IF RT (36–40 Gy)	168	**88**	92	—	—	1/0.01
(Noordijk EORTC-H7F 2006)		*P* = .0113				

STLI	272	74 (EFS 5 y)	92 (10 y)	—	—	0/2
3MOPP/ABV + IF RT (36 Gy)	270	**98**	**97**	—	—	0/2
(Ferme EORTC/GELA-H8F 2007)		*P* < .001	*P* < .001			

4ABVD + STLI RT	136	93 (12 y FFP)	96	—	—	1.5/3
4ABVD + IF RT		94	94	—	—	—
(Bonadonna Milan 2004)						

4ABVD + IF RT	1370	93 (FFTF 5 y)	97	24	52	
2ABVD + IF RT		91	97	**15**	**33**	
ABVD + IF RT (30 Gy)		93	98		8.7	
ABVD + IF RT (20 Gy)		93	93		**2.9**	
(Engert GHSG-HD10 2009)						

STLI	64	88 (5 y FFP)	100	—	—	—
4–6ABVD	59	87	97	—	—	—
(Meyer NCIC/ECOG HD6 2005)						

6ABVD + IF/EF RT	76	86 (5 y FFP)	97			
6ABVD	76	81	90			
(Straus MSKCC 2004)						

**Table 4:** Trials of treatments for early stage unfavourable Hodgkin's lymphoma. Numbers in bold are statistically significant with *P* values where significant. ^*∗*^Treatment discontinuation due to haematological intolerance. Abbreviations: OS: overall survival; EF RT: extended-field radiotherapy; IF-RT: involved-field radiotherapy; STLI: subtotal lymphoid irradiation; Gy: gray; ABVD: doxorubicin, bleomycin, vinblastine, and dacarbazine; EBVP: epirubicin, bleomycin, vinblastine, and prednisolone; MOPP/ABV: mechlorethamine, vincristine, procarbazine, prednisolone, doxorubicin, bleomycin, and vinblastine; COPP/ABVD: cyclophosphamide, vincristine, procarbazine, and prednisolone/ABVD; BEACOPP: bleomycin, etoposide, doxorubicin, cyclophosphamide, vincristine, procarbazine, and prednisolone.

Treatment regimen (author/trial)	No. of Pts	Outcome (%, measure, time)	OS (%)	Haem toxicity (% grade III-IV)	Second malignancy (haem/solid, %)
3 MOPP + Mantle RT + 3 MOPP	165	77 (FFP 6 y)	85	14.5	—
3 ABVD + Mantle + 3 ABVD	151	**88**	91	7.3*	—
(Carde EORTC H6U 1993)		*P* = .01			

6EBVP + IF RT (36 Gy)	194	68 (EFS 10 y)	79	—	4/5
6 MOPP/ABV + IF RT	195	**88**	**87**	—	2/2
(Noordijk EORTC H7U 2006)		*P* < .001	*P* = .0175		

2ABVD + STLI	139	**95** (FFP 5 y)	92	—	—
4–6ABVD	137	88	95	—	—
(Meyer NCI-C/ECOG HD6 2005)		*P* = .004			

6 MOPP/ABV + IF RT (36 Gy)	336	82 (EFS 10 y)	88	—	2/2
4 MOPP/ABV + IF RT (36 Gy)	333	80	85	—	3/3
4 MOPP/ABV + STLI	327	80	84	—	2/2
(Ferme EORTC-GELA H8U 2000)					

4 COPP/ABVD + EF RT (30 Gy) + Bulky 10 Gy	532	86 (FFTF 5 y)	91	5.8	2.2/2.3
4 COPP/ABVD + IF RT (30 Gy) + Bulky 10 Gy	532	84	92	**2.5**	1.2/1.7
(Engert GHSG HD8 2003)				*P* < .001	

**Table 5:** Trials comparing MOPP- and ABVD-based regimens for the treatment of advanced stage Hodgkin disease. Numbers in bold are statistically significant with *P* values where significant. *Febrile neutropenia. Abbreviations: OS: overall survival; MOPP: mechlorethamine, vincristine, procarbazine, prednisolone; ABVD: doxorubicin, bleomycin, vinblastine, and dacarbazine; MOPP/ABV: mechlorethamine, vincristine, procarbazine, prednisolone, doxorubicin, bleomycin, and vinblastine.

Treatment regimen (author/trial)	No. of Pts	Outcome (%, measure, time)	OS (%)	Haem toxicity (% grade III-IV)	Second malignancy (haem/total, %)
MOPP	198	54 (FFP 20 y)	48	—	<2/− (10 y)
(Longo 1986)					

MOPP	43	36 (FFP 8 y)	64	—	—
MOPP/ABVD alternating	45	**65**	**84**	—	—
(Bonadonna 1986)		*P* < .005	*P* < .06		

6–8 MOPP	123	50 (FFS 5 y)	66	Increased	—
MOPP/ABVD alternating ×12	123	61	73	—	—
6–8 ABVD	115	65	75	—	—
(Canellos 1992)					

MOPP/ABVD alternating	211	67 (FFP 10 y)	74	—	—
MOPP/ABVD hybrid	204	69	72	—	—
(Viviani 1996)					

MOPP/ABV hybrid	153	71 (FFS 5 y)	81	18*	—
MOPP/ABVD alternating	148	67	83	**7**	—
(Connors 1997)				*P* = .0001	

MOPP/ABV	419	66 (FFS 5 y)	81	75	3/7
ABVD	433	63	82	**64**	**0.005**/4
(Duggan 2003)				*P* = .001	*P* = .011

**Table 6:** Trials of treatments currently under investigation for the treatment of advanced stage Hodgkin disease. Numbers in bold are statistically significant with *P* values where significant. Abbreviations: OS: overall survival; RT: radiotherapy; MOPP: mechlorethamine, vincristine, procarbazine, and prednisolone; ABVD: doxorubicin, bleomycin, vinblastine, and dacarbazine; COPP/ABVD: cyclophosphamide, vincristine, procarbazine, and prednisolone/ABVD; BEACOPP: bleomycin, etoposide, doxorubicin, cyclophosphamide, vincristine, procarbazine, and prednisolone; CEC: cyclophosphamide, lomustine, vindesine, melphalan, prednisolone, epidoxorubicin, vincristine, procarbazine, vinblastine, and bleomycin; Stanford V: doxorubicin, vinblastine, mechlorethamine, vincristine, bleomycin, etoposide, and prednisolone; MOPPEBVCAD: mechlorethamine, vincristine, procarbazine, prednisolone, epidoxorubicin, bleomycin, vinblastine, lomustine, doxorubicin, and vindesine; MDR ChIVPP: (chlorambucil, vinblastine, procarbazine, and prednisolone) alternating either with PABIOE (prednisolone, doxorubicin, bleomycin, vincristine, and etoposide) or EVA (etoposide, vincristine, and doxorubicin); ASCT: autologous stem cell transplant.

Regimen (author, trial)	No. of Pts	CR (%)	Outcome (%, measure, time)	OS (%)	RT (%)	Haem toxicity/infections (gr III-IV, %)	Second malignancy (haem/total, %)
6–8 MOPP	123	67	50 (FFS 5 y)	66			
MOPP/ABVD alternating ×12	123	83	61	73			
6–8 ABVD	115	82	65	75			
(Canellos 1992)							

8COPP/ABVD	261	85	64 (FFTF10 y)	75	64	71/3	**0.4**/5.3
8BEACOPP	469	88	70	80	71	73/**16**	2.2/7.9
8EscBEACOPP	466	96	**82**	**86**	71	**98**/**22**	3.2/6.5
(GHSG HD9 2003)			*P* < .0001	*P* = .005			*P* = .03

6ABVD	99	70	65 (FFS 5 y)	84	46	**34**/2	0/1
BEACOPP 4 escalated, 2 baseline	98	81	**78**	92	44	54/**14**	0/1
6CEC	98	69	71	91	43	48/4	1/2
(Federico—GISL HD2000—′09)			*P* = .036 versus ABVD			*P* = .016/*P* = .003	

8 × BEACOPP-14 ± RT	94	94	97 (FFTF 3 y)	90	70	75/12	1/1
(Sieber GHSG Phase II 2003)							

6ABVD	122	89	78 (FFS 5 y)	**90**	76	25/1	0/0
3Mod Stanford V	107	76	**54**	82	71	29/0	1/2
6MOPPEBVCAD	106	94	81	89	50	**51**/**14**	4/4
(Gobbi Italian Intergroup 2005)			*P* < .01				

ABVD	261	46	76 (PFS 5 y)	90	53	—	0.004/0.02
Stanford V	259	44	74	92	73	—	0.012/0.02
(Hoskin UK NCRI 2009)							

ABVD ± RT	406	68	75 (3 y)	90	37	56	0/−
MDR ± RT	401	67	75	88	49	**65**	−/−
(Johnson UK LY09 2005)							

8ABVD	80	89	82 (FFS 5 y)	88	—	—	—
4ABVD + ASCT	83	92	75	88	—	—	—
(Federico EBMT HD01 2003)							

**Table 7:** Summary of outcomes of nonrandomised trials of salvage treatments in relapsed and refractory Hodgkin's lymphoma, arranged in descending order of ORR. Abbreviations: CR: complete remission; PR: partial remission; ORR: overall response rate; TRM: treatment-related mortality; DHAP: dexamethasone, cytarabine, and cisplatin; ICE: ifosfamide, carboplatin, and etoposide; IVE: ifosfamide, etoposide, and epirubicin; IV: ifosfamide, vinorelbine; Mini-BEAM: BCNU, etoposide, cytarabine, and melphalan; Dexa-BEAM: dexamethasone, carmustine, etoposide, cytarabine, and melphalan; ESHAP: etoposide, methylprednisolone, high-dose cytarabine, and cisplatin; ASHAP: doxorubicin, methylprednisolone, high-dose cytarabine, and cisplatin; MINE: mitoguazone, ifosfamide, vinorelbine, and etoposide; GVD: gemcitabine, vinorelbine, and pegylated liposomal doxorubicin; GDP: gemcitabine, dexamethasone, and cisplatin.

Regimen	No. of	CR %	PR %	ORR %	Neutropenia	Thrombocytopenia	Vomiting	TRM %
	Pts	(95% CI)	(95% CI)	(95% CI)	% Gr 3/4	% Gr 3-4	% Gr 3-4	(95% CI)
DHAP [54]	102	21	68	89	88	69	26	0
		13–29	59–77	83–95				0–4
ICE [55]	65	26	59	88	—	—	—	0
		16–39	46–71	74–92				0–5
IVE [56]	51	61	22	84	100	—	—	0
				71–93				
Mini-BEAM [57]	55	51	33	84	86	60	—	2
		35–63	21–47	69–91				0.1–10
IV [58, 59]	47	45	38	83	65	0	2	—
		30–60	25–54	69–92				
MINE [60]	157	—	—	75	—	—	—	5
		64–84						
ASHAP [61]	56	34	36	70	100	—	—	0
GVD [62]	91	19	51	70	68	28	1	1
				60–80				
GDP [63]	23	17	52	69	9	13	13	0
		5–39	31–73	52–87				0–15
Dexa-BEAM [64]	55	31	29	60	>90	>90	—	5
				46–73				1–9

**Table 8:** Registry data of outcomes of myeloablative allogeneic transplants in relapsed and refractory Hodgkin's lymphoma. Abbreviations: Sib/UD: Sibling/unrelated donor; PFS: progression-free survival; OS: overall survival; TRM: treatment-related mortality; IBMTR: International bone marrow transplant registry; EBMT: European Group for Blood and Marrow Transplantation; FHCRC: Fred Hutchinson Cancer Research Center; JHOC: Johns Hopkins Oncology Center.

Study (group, author)	No. of pts	Sib/UD	Median age (range)	PFS (%, years)	OS (%)	TRM (%)
IBMTR	100	100	28	15 (3 y)	21	61
(Gajewski 1996)			(12–44)			
EBMT	45	45	29	15 (4 y)	25	48
(Milpied 1996)			(15–42)			
FHCRC	53	50/3	29	18 (5 y)	21	49
(Anderson 1993)			(10–55)			
JHOC	53	53	28	26 (10 y)	30	43 (total)
(Akpek 2001)			(13–52)			
EBMT	167	145/12	24	16 (4 y)	25	52
(Peniket 2003)			(7–57)			

**Table 9:** Registry data of outcomes of reduced intensity conditioned (RIC) allogeneic transplants in relapsed and refractory Hodgkin's lymphoma. Numbers in bold are statistically significant with *P* values where significant. *Antithymocyte globulin given to unrelated donor recipients. Abbreviations: Sib/UD: sibling/unrelated donor; PFS: progression-free survival; OS: overall survival; TRM: treatment-related mortality; M: melphalan; F: fludarabine; A: alemtuzumab; MDACC: MD Anderson Cancer Center; EBMT: European Group for Blood and Marrow Transplantation.

Study (group, author)	No. of pts	Sib/UD	Median age (range)	PFS (%, years)	OS (%)	TRM (%)
RIC	52	−/−	30	42 (2 y)	56	17
(EBMT Robinson 2002)			15–53			
MF-A	49	31/18	32	39 (4 y)	56	16 (2 y)
(UK Peggs 2005)			18–51			
MF*	40	38/2	31	32 (2 y)	48	25 (1 y)
(Spain Alvarez 2006)			16–53			
MF*	58	25/33	32	32 (2 y)	64	15
(MDACC Anderlini 2008)			19–59			
Myeloablative	79	70/9	27 (11–60)	20 (5 y)	22	48 (3 y)
RIC	89	77/12	26 (5–61)	18	**28**	**24**
(EBMT Sureda 2008)					(.04)	*P* = .003

**Table 10:** Comparison of early phase studies of novel agents in relapsed and refractory Hodgkin's lymphoma. Abbreviations: PR: partial response; CR: complete response; ORR: overall response rate; IL2-R: interleukin 2 receptor; HDAC: histone deacetylase; mTOR: mammalian target of rapamycin.

Novel agent (author)	Target	No. of Pts	Route	Phase	PR (%)	CR (%)	ORR (%)
SGN30 (Forero-Torres 2009)	CD30	38	IV	II	0	0	0
MDX060 (Ansell 2007)	CD30	47	IV	I/II	4	4	8
SGN35 (Younes 2008)	CD30	44	IV	I	14	25	39
SGN35 (Bartlett 2009)	CD30	17	IV	I	6	41	47
CHT25 (Dancey 2009)	IL2-R radioimmunotherapy	9	IV	I	33	33	66
MGCD0103 (Younes 2007)	HDAC class I	21	Oral	II	29	9	38
Panobinostat (Dickinson 2009)	Pan HDAC	13	Oral	I/II	54	0	54
Panobinostat (Younes 2009)	Pan HDAC	27	Oral	II	15	4	19
Vorinostat (Kirschbaum 2007)	Pan HDAC	25	Oral	II	4	0	4
Everolimus (Johnston)	mTOR	19	Oral	II	42	5	47
Bortezomib (Younes 2006)	Proteasome	14	IV	II	7	0	7
Bortezomib (Blum 2007)	Proteasome	30	IV	II	0	0	0
Rituximab (Rehwald 2003)	CD20	14	IV	II	57	29	86
Rituximab (Younes 2003)	CD20	22	IV	II	18	5	22
Lenalidomide (Boll 2009)	Immunomodulator	12	Oral	II	23	8	50
Lenalidomide (Kuruvilla)	Immunomodulator	17	Oral	II	47	6	53
